# Regular Meeting of the Fulton County Medical Society, Thursday, April 6, 1916

**Published:** 1916-04

**Authors:** 


### REGULAR MEETING OF THE FULTON COUNTY MEDICAL SOCIETY, THURSDAY, APRIL 6, 1916.

A case of Amoebic Dysentery with abscess of liver in which the patient was operated on and upon recovery was reported by Drs. LeRoy W. Childs and C. W. Strickler.

Dr. Stewart R. Roberts presented a case on "Decrescent Arteriosclerosis with Hemiplegia and Hemataxia." The history of the case is as follows:

Age 59. Admitted Dec. 20, 1915. Complaint: Unconscious on admission.

F. H. Maternal G. F. died in asylum. A twin sister had three strokes of paralysis and died 6 years ago at the age of 53. Mumps, measles and w. c.; typhoid at 20. Two years ago was run over by automobile and left hip injured. Five weeks later suffered a left hemiplegia (motor) and in few weeks had nearly as good use as ever of left side. He has been nervous since. Syphilis and gonorrhea denied. No drinking or smoking. Has eaten largely. Digestion good, bowels open.

P. I. On Dec. 20, 1915 he suffered a severe headache, and later marked vertigo, room turned dark, lapsed into unconsciousness. Dec. 20, B. P. 140-90-50. The next day he woke to find himself in the hospital. Headache continued, hiccoughs and nausea. During Jan. 1916, he lay helpless in bed with alternating periods of unconsciousness, nervous, could not swallow easily, and was fed by rectum. Jan. 13, severe frontal headache and nausea.

P. E. Feb. 1, Can open mouth only slightly, pulls down chin; cannot extrude tongue but pulls it out. Tongue deviates to left, gag reflex gone, uvula to left. Apex beat 5th space normal, sounds weak. Arteries small and hard. Left arm B. P. 120-80-40, right arm B. P. 140-95-45. Respirations vary from 16 to 24. Temperature rises to 106.6. Pulse 80 to 108. Abdomen, negative. Nervous system: complete motor and sensory paralysis of entire left side except the left occipito-frontalis contracts. Elbow reflexes increased, right elbow spastic, tapping righ biceps causes clonic contraction and twitching of right hand; left fingers flexed over thumb in state of tonic spastic contraction. and tonic flexion left hand. Extension of left fingers possible if thumb is abducted. If thumb if abducted, fingers close like trap. Left cremasteric absent. Epigastrics present. Left knee jerk normal, right plus. Left ankle clonus, right ankle spastic. No Babinski, no Oppenheim. Entire left side negative to pain, pressure, heat, cold and muscle sense. Pronounced hemi-taxia, both motor and static. Pronounced proximo-ataxia and left acro-ataxia.

Cranial Nerves:

1st nerve: Complete left anosmia.

2nd nerve: Left eye nearly complete blindness, no reaction to L. and L., cannot distinguish man 12 ft. or finger 1 ft. distant. Atrophy of disk left eye, temporal side, outline of disk indistinct. Vessels very tortuous, corkscrew arteries. Probably descending atrophy.

3rd and 4th, 6th nerves: Left pupil dilated. All muscles of left eye partially paralyzed.

5th nerve: Paralysis of muscles of mastication, inability to open mouth, loss of sensation entire left face including the conjunctiva, taste anterior two-thirds lost.

7th nerve: Partial paralysis, cannot shut eye, but can keep it shut after closing it with finger.

8th nerve: Absolute deafness in left ear. Right ear, watch tick heard at three inches.

9th nerve: Complete paralysis, no sensation of taste posterior one-third tongue.

10th nerve. Partial paralysis, difficulty in swallowing.

Rapid pulse 90, respiration varys from 8 to 28, choked at times even with water.

11th nerve: Complete paralysis.

12th nerve: Partial paralysis, hemiatrophy of left tongue increasing.

March 22, tongue now deviates to right. Urine: negative. Hemoglobin 70%. Repeated W. aR. on blood) negative. Spinal fluid WaR. negative; cell count 4. No increase in globulins. Treatment: Rest, massage, early rectal feeding, iodids and mercury. On Feb. 21, patient was put on increasing doses of nux vomica and showed marked improvement.

P. C. March 22, patient can walk poorly, hemiaplegic gait, can sit up, speech stammering, mind good; sight, hearing, swallowing and strength poor; marked hemi-ataxia continues. pulse 98, no nausea.

Summary: Decrescent arterio-sclerosis, two attacks of cerebral hemorrhage, complete left motor and sensory hemiplegia, partial or complete paralysis of all cranial nerves.

Diagnosis: Cerebral hemorrhage in region of right internal capsule involving both motor and sensory knee and optic thalamus, partial or complete paralysis of all cranial nerves, decrescent arterio-sclerosis, senile kidney.

Dr. Rufus T. Dorsey exhibited a case of "Hemiplegia in a Young Girl," following an abdominal operation.

Drs. J. L. Campbell and T. B. Armstrong presented a case of "Traumatic Epilepsy." The history of the case is as follows:

Hamp Wright, colored. Was admitted to Grady Hospital in February, 1915. Injury to head.

While at work on a car track was struck by fellow worker on the left side of the head with the sharp end of a pick. The skull was fractured for an area about 3x2½ inches. The pick penetrated the bone a little below the parietal eminence, passed through the dura and into the brain substance for an inch and a half. There was crushed bone, cinders and grit between the bone and the dura. A few strands of hair, crushed bone and a large quantity of clotted blood were beneath the dura.

All the fragments of loose bone were removed. A piece of brain tissue 1 in. deep, one-fouth to one-third inches wide and about the same thickness was found loose and removed. The dura was opened and a large flat clot washed out and the wound otherwise cleansed as well as possible. The dura was closed, except at the point of entrance of the pick where a rubber dam was inserted and carried to the bottom of the brain wound. The scalp was also closed except at the point of exit of the drain.

The patient made an uneventful recovery and was well for about six months when he began to have fits of an epileptic character. These continued to grow worse and more frequent.

Readmitted to the hospital March 8, 1916: A fit described by a fellow patient was about as follows: "Contractions begun in the right hand, extended to the shoulder and whole right side. Then the whole body seemed to be involved. There is a short period of sleep and heavy breathing.

Operation: College, 9 a. m. March 14, 1916. An incision was made around the upper margin of the opening in the skull and the scalp reflected downward. The dura was opened just in front of a firm scar which appeared at the point of entrance of the pick. Slight adhesions were found between the dura and brain surface which was broken up to the margin of the skull opening and the dura reflected forward. The dura adhesions were cut and the posterior half of the dura reflected backward. All adhesions around the margin were freed and the dura cut away and the margin turned out and stitched to the pericranium.

A thin celluloid plate was cut just a little larger than the opening in the skull and tacked to the margin of the dura with small carpet tacks and the scalp wound closed. Bleeding was controlled in the scalp by a tourniquet around the head below the incision. Bleeding from the adhesions was moderately free but was controlled by pressure with cotton sponges wet with adrenalin chloride 1-1,000. The operation consumed about 2 hours. The patient's condition was good when returned to the hospital. Recovery was complete.

